# Home care in dementia: The views of informal carers from a co-designed consultation

**DOI:** 10.1177/1471301221990504

**Published:** 2021-02-03

**Authors:** Elizabeth L Dalgarno, Vincent Gillan, Amy Roberts, Jean Tottie, David Britt, Cecilia Toole, Paul Clarkson

**Affiliations:** Social Care and Society, 5292 University of Manchester, Manchester, UK; Social Care and Society, 5292 University of Manchester, Manchester, UK; Social Care and Society, 5292 University of Manchester, Manchester, UK; Family Carer, Together in Dementia, Liverpool, UK; Family Carer, Manchester, UK; Family Carer, Sight and Mind, Merseyside, UK; Social Care and Society, 5292 University of Manchester, Manchester, UK

**Keywords:** home care, dementia, caregiving, informal carers, personalised care, continuity of care, recognition/status of carers, value of care, time and task

## Abstract

**Background:**

In the United Kingdom, there is a current priority for high-quality dementia care provided at home. However, home care or domiciliary care is an area where problems have been reported, in terms of a lack of consistency, coordination and appropriate responses to the specific needs of those with dementia. The views of informal carers, who often must respond to these problems when supporting relatives, are crucial in shedding light on the issues and in seeking to promote solutions.

**Methods:**

This study explored the views of informal carers of those with dementia concerning home care, through a consultation using an electronic survey. The survey questions were designed by informal carers, through a public involvement group within an existing programme of dementia research. The survey elicited responses from 52 informal carers in 2017/18. The data were analysed qualitatively using framework analysis.

**Findings:**

Carers’ views focused on the need for investment into meaningful personalisation, recognising the value of providing care and valuing formal carers, systemic failings of care coordination and provision and the importance of ongoing collaboration and care planning.

**Conclusion:**

Based on a framework drawn from the views of informal carers themselves, this study articulated issues of concern for home care and its delivery for people with dementia. Attempts should be made to make dementia home care more consistently personalised, inclusive and collaborative with informal carers and key others involved. Further areas to explore include working conditions of formal carers and current models utilised in homecare provision.

## Background

The UK Government has stated that by 2020 it wants to see an increase in the numbers of people with dementia being able to live at home. It wants to see a greater provision of innovative and high-quality dementia care provided at home, suitable to the individual needs of the person with dementia, their carers and families. For this to be possible, the Government has stated that there must be greater efforts to make home care an attractive profession (Dementia 2020 Challenge: 2018 Review Phase 1, Department of Health and Social Care, 2019).

In the United Kingdom, the remit, scope and forms of service delivery in adult social care are complex (Department of Health and Social Care (DHSC), 2018); however, most home care is funded by directors of adult social care, within local authorities (LAs) who then commission care services from a mixture of statutory, private and voluntary (non-profit) organisations. Nevertheless, a lack of data on its content has been reported (Knapp et al., 2007). Owing to the lack of specified information describing home care approaches in prior research, a relatively precise definition of home care, particularly as it pertains to people with dementia, was warranted to guide this study. Home care has been defined for the purpose of community care audit as:

“…services that assist the service user to function as independently as possible and /or continue to live in their own home. Services may involve routine household tasks within or outside the home, personal care of the user or respite care in support of the user’s regular carers. It excludes services such as day care, meals, transport and equipment.” (Office for National Statistics, 2005, p.1).

The core elements of home care, according to this definition, may therefore be regarded as help with personal care including bathing, dressing, transferring in and out of bed or a chair; meal preparation; medication management; light housework; managing finances; shopping and accompanying a service user outside the home (Equality and Human Rights Commission, 2011). This definition also includes respite care, but that provided by a (formal) carer sitting in the home to support the older person (so-called ‘in-home care’) rather than that which transfers the service user out of the home to give the informal carer a break (‘day care’). The term ‘informal carer’ is often disputed; however, it has been adopted here as the least problematic and with the broadest consensus in the United Kingdom (see Beesley, 2006). These elements of care can be offered to people with complex needs as well as to those requiring less intensive help with relatively simple tasks, for example domestic aid for frail older people (Genet et al., 2011). A recent, international, narrative review on homecare services, specifically for people with dementia (Low & Fletcher, 2015) defined home care as:

“…non-medical support to people in their own homes, also known as home care, community care, domiciliary care, social care, or in-home care. We exclude home healthcare which is provided by medical, nursing and allied health professionals as these services differ from home (non-health) care, typically focusing on improving or maintaining health rather than supporting function.” (p.1593)

The crucial aspects to this definition are that home care supports function rather than treatment for health concerns and is provided by non-medical or non-health practitioners. These two definitions of home care are used to guide this study.

Existing research has examined the experience of being an informal caregiver to a person with dementia and service utilisation, but there is limited research using a qualitative approach or exploring this for community-dwelling caregivers (Cascioli et al., 2008; Crellin et al., 2014; Fonareva & Oken, 2014; Karlsson et al., 2015; Lethin et al., 2016; McCabe et al., 2016; Neville et al., 2015; Peacock, 2013; Quinn et al., 2010). Within the extant literature, problems have been reported in terms of a lack of consistency and coordination of formal carers for people with dementia and appropriate responses to the specific needs of those with dementia (McCabe et al., 2016).

This study aims to examine formal home care for community-dwelling informal carers and individuals with dementia by exploring the views of informal carers, who often must respond to these problems when providing support. Informal care will be defined here as care ‘provided by non-professionals who are not compensated for their service’ (Mentzakis, et al., 2009, p. 283). This research is now needed to shed light on these issues and is necessary to facilitate and promote appropriate solutions.

## Methods

The study was intended to inform the research question derived from the wider study of dementia home care, of which it was a part, namely what is appropriate home care for people with dementia and how does it fit with the preferences of informal carers? Ethical approval was granted by the NHS Research Ethics Committee, North West, Haydock (14/NW/1044; 17 July 2014), as part of a wider programme of research on home support in dementia (HoST-D). The research was designed at the outset to be co-designed by informal carers who had experienced the provision of home care and researchers, and it was decided that the research question could best be answered by using qualitative methods (Bryman & Burgess, 1994). Inclusion criteria reflected this and were broadly to have been a carer of someone with dementia, now or previously, or other carer interested in provision of care for people with dementia. We wished to explore, in-depth, a number of emerging themes and issues, concerning the provision and delivery of home care specifically for people with dementia, from the perspective of family members offering support. Issues from the point of view of providers of home care are being explored in another study. For this study, a survey tool with relatively open-ended questions was designed to capture issues of concern to informal carers. Often, open-ended surveys allow participants to provide answers to general questions that are distinctive and provide rich data that may reveal issues not captured by closed questions. In this respect, the intention of a qualitative survey is not to provide indications of the distribution of phenomena amongst groups of participants but is directed at determining the diversity of the topic of interest (Jansen, 2010).

### Developing the survey

A dynamic, iterative process was used in designing the survey from which data to examine issues around home care for informal carers could be derived (Bryman & Burgess, 1994). First of all, issues with home care for informal carers were explored in a public involvement group as part of an existing dementia research study, the HoST-D Programme, for which the public involvement aspect has already been reported (Giebel et al., 2019). Five existing or former carers of people with dementia attended the group, and the discussion was opened up with a description of home care in England and a brief presentation of some of the issues. It was not intended, however, to constrain issues that it would be possible to discuss, thus possibly prejudicing carers’ opinions. Therefore, the group were directed that no issue was ‘off the table’ and that they could be free to raise any issue, from their experience, that they felt was pertinent. The discussion was transcribed directly, verbatim, by one researcher (VG) and, subsequently, the notes of the discussion were fed back to members of the group for them to signal that they represented an accurate record of the session. Secondly, three researchers (PC, VG and AR) met to draw out global themes from the discussion notes that could act as a guide to developing general questions to be included in the survey. This process included one researcher (AR) who was not involved in the original discussion of the public involvement group of carers. Six broad themes emerged, of interest to carers, as to the elements important in signalling ‘good’ home care specifically for people with dementia. These were cultural sensitivity (ability to speak the same language as the person with dementia), timeliness of care (adequate time with the person at each session to meet his/her needs/arriving promptly at the agreed time for each session), the relationship element (building a close working relationship with the person or with the person’s family), care delivery/care planning (consistency in care provision; knowing in advance full details of person’s needs/sharing responsibility with one trusted colleague/having sole responsibility for the home care of an individual’s needs), formal qualifications/experience/working practices (possession of formal care qualification; previous experience of caring for one or more people with dementia; being monitored/probation period) and emotional aspects (feeling compassion for the individual/having empathy with them). In a third stage, these themes elicited from the discussion notes were fed back to the carers group and they were asked to devise broad questions that could touch on them. These broad questions were then taken forward in constructing the survey tool. The questions included in the tool are listed in Box 1.

Box 1: Questions included in the survey tool

Questions

1. From your experience do the home carers recognise the person’s individual needs (e.g., cultural, faith and disability)?
2. Are carers able to spend enough time with the person with dementia? If not, explain the difficulties.
3. How have carers developed a relationship with the person with dementia? How have they understood their life history and specific likes/dislikes?
4. How has care been delivered? Do you think care workers are aware of the care plan? How are you (as the family carer) included in what happens?
5. Are care workers qualified for the job? Have they sufficient knowledge and skills required?
6. Do you feel the care worker made a connection with the person? How do you think the worker showed compassion or empathy for the person with dementia?
7. Lastly, have you any other comments to make? What do you think constitutes good home care for people with dementia?

The survey was then designed using SurveyMonkey which provides a secure, privately accessible questionnaire for completion by participants, with the ability for data to be exported to Excel/CSV files for use in data analysis. The broad questions were included in the survey as drop-down boxes, permitting respondents to feely enter text to respond to each question or raise issues. Also included in the tool were structured questions with multiple-choice responses to elicit data on participants’ age range, gender and status (whether they were an existing or former carer of a person with dementia). However, the tool was designed to collect completely anonymised data and thus no questions concerning potential personal data, such as geographic location (e.g. postcode) or name, were included. Once the survey was designed, it was sent to each member of the public involvement group for their comments, and they were asked, if they wished, to complete the survey to test if their answers could be viewed correctly when exported as response data. These data were not used in the final analysis.

### Data collection

Following the process above, the survey was then hosted on the website of TIDE – Together in Dementia Everyday (http://tide.uk.net/) – a charity that has a UK-wide network of carers of people with dementia as members. Any interested member was invited to complete the survey via a secure web link. Completion of the survey was assumed to signal consent and participants were informed of this at the outset through an outline description of the study on the website. Responses to the survey were exported to Excel through a secure (password protected) portal in readiness for data analysis.

### Data analysis

Data were analysed qualitatively by emergent themes, using framework analysis (Ritchie & Spencer, 1994). The framework approach was viewed as particularly useful as it is designed for thematic analysis that aims to examine policy issues, such as what are the appropriate ways to deliver home care, specifically for people with dementia. Two researchers (ED and AR) undertook the analysis with constant comparison of emerging themes. Data were examined, recorded and categorised in accordance with key issues and themes. This involved a five step process of familiarisation, identifying a thematic framework, indexing, charting and mapping and interpretation (Ritchie & Spencer, 1994). Analysis was iterative and ongoing discussion between the two researchers ensured reliability of the findings.

### Findings

Table 1 shows the characteristics of participants who responded to the carers’ survey. Fifty-two carers participated and gave their views.

**Table 1.** Circumstances of carers completing the survey (*n* = 52).

Circumstance	*N*	%
Who completed?		
Former carer	17	33
Current carer	29	56
Interested person not caring for someone with dementia	5	9
Missing	1	2
Age		
30–49	8	15
50–60	17	33
61 and over	27	52
Gender		
Male	12	23
Female	40	77
How long caring?		
Less than a year	1	2
More than a year	32	62
Not caring at the moment	16	31
Missing	3	5

Situated within the six broad themes (identified above) used to develop the survey questions, the following key themes were identified from the survey answers as pertinent to high-quality dementia home care:

1. Investment into developing meaningful personalisation
2. Recognising the value of providing care and valuing formal carers
3. Systemic failings of care coordination and provision
4. The importance of ongoing collaboration and care planning

1*. Investment into developing meaningful personalisation*: Family members and informal carers described personalisation of care for people with dementia as challenging without due time devoted by formal carers to developing meaningful relationships. This challenge was exacerbated by the specific nature of dementia as particularly demanding and changeable or fluid, thus requiring familiarity with the individual as well as knowledge and experience of the condition:

Good home care is very demanding and requires experience as well as knowledge and skills. Person centred care is difficult to achieve in any circumstance but made doubly difficult by the widely differing impact of dementia on people and the often ‘thankless’ nature of the ongoing care needs.

Time spent before the actual need for care. Ideally, I would have started a carer visiting first and developing a relationship, then gradually introduced the carer helping me with care then taking over. Unfortunately, as it was a crisis point this couldn't happen, but the relationship building is essential for the person living with dementia and the family carer.

Discontinuity of the relationships between the person with dementia and the formal carers created further challenges to developing meaningful personalisation. Families/informal carers consistently reported that a connection between the individual and formal carer could not be achieved when the formal carers were frequently changed. This was mostly perceived as poor care coordination (presented in theme 3), rather than the carers lacking personal motivation to connect with the service user. Accessing the person with dementia’s likes, dislikes and *past life* to enable personalisation, therefore, became unattainable:

The high number of different home carers visiting made it hard for the workers to develop a relationship and made it distressing, sometimes, for the person with dementia as they couldn’t always remember the workers so it felt like a stranger visiting

Not all home carers have that opportunity as unfortunately they are moved around clients to fill gaps in rotas. The best situation is where the same regular carers visit so a meaningful relationship can be built up over time.

Conversely, where families reported consistency of formal carers, this meant they ‘*could get to know an individual much better and therefore be able to understand their world more easily’.* Meaningful connectedness was frequently referred to as a desired element, highly valued by families:

The care worker has a very close connection with my husband having worked with him for 2 years. He is aware of his feelings and reactions to what is happening. I am in a unique position having just one carer and he has been with us for a long time.

*‘Good care’*, was frequently characterised by continuity of formal carers, care that was reactive and engaging and highly personalised to the individual at an emotional, social and cultural level:

The carer has worked well with my husband, he has taken it slowly and reacts to his needs and preferences and remembers them.

One of the home carers tried to cook traditional food. Another one of the carers tried to learn words of the language that the person they were caring for spoke.

I had one private carer who gave us support over the years and she became and still is a friend. The carers from agencies very seldom have an opportunity to develop an in-depth knowledge of their clients, particularly when the client has dementia and has no awareness of their past life.

The functional aspects of care alone being met did not amount to a meaningful interaction; rather, it was meaningful communication that created the connectedness between the carers and person with dementia:

One carer Dad had got on his wavelength and they used to sing old army songs and have a good laugh whilst she did his chores. She was the exceptional carer worth her weight in gold.

 2*. Recognising the value of providing care and valuing formal carers*: was a key theme for participants. In particular, the skills and personal qualities of formal carers such as ‘*warmth*’ and ‘*kindness*’ were described as pivotal to the development and maintenance of optimal care provision, as this reassured families that the right people were caring for their loved one. Personal qualities and attitudes of formal carers were equally weighted with formal qualifications as essential to high-quality care for most participants, although many stated they would also welcome more of the latter:

There are a few rare Home care workers who have the correct skills, compassion and empathy. I believe that this has come about because they are truly dedicated to delivering personalized quality care.

Again, personality of the carer came into it. Empathy went a long way. Mum knew when she was being patronised. She loved a laugh.

Participants discussed a broader absence of recognition for the value of care workers, reflected by a perceived lack of this and investment from society and employers into their skills, training and supervision, with minimal consideration given to the demanding nature of their roles. Participants frequently described formal carers as *‘undervalued’* and *‘underpaid’*:

Stop using negative terms like low paid unskilled workers. Recognise their potential and invest in them.

There is a vast difference in skills and training from various agencies. No standardised training programme no career structure. I think that many of them have the right attitude and compassion but don’t have the resources to go with this. They have the potential to become a very skilled workforce but need to be invested in and valued just like family carers.

They did not receive sufficient training for the level of responsibility they had in relation to the dependency of the client. Often family carers of people with dementia are elderly themselves and possibly with health issues. Care workers have to face some difficult situations.

This lack of recognition was implicitly linked to broader employment issues such as ‘*zero hours contracts*’ and the perceived public undervaluing of those living with dementia, as many participants felt their family members’ needs were disregarded by society and of lesser importance than others in society:

I feel that for the majority of people applying for these positions it is an easy job to get. There must be some who have knowledge, skill and experience but I have not come across them. I find it hard to believe that such people could be in charge of children.

Their employer only allows a half hour slot. Not enough people willing to do Care. Poor working conditions for paid carers zero hrs contract. Minimum pay, not being with the same person on each visit. Funding in the community (care package) is inadequate to pay for the correct amount of time.

Linked to this was the insight from participants that they often perceived formal/paid carers as lacking an in-depth knowledge and training specific to dementia. This was expressed as concerning and saddening for family members/informal carers, with some attempting to address this themselves:

Often carers new to my husband were not aware that he had Early Onset Dementia. I would have to inform them and go through a 'This is Me' write up to inform him of his preferences and understanding of his dementia and as he has Lewy Body Dementia explain the difference between this and other dementias.

Many care workers are experienced and have no knowledge of dementia and the needs. Just a basic online course completed at best.

 *Participants described* (3) *systemic failings of care coordination and provision*: characterised by an approach that was stifling to good care, burdensome and restrictive to families and informal carers. They conveyed a desire for the systems to be more inclusive of families and stated this would facilitate a more beneficial approach for informal carers and the person with dementia. Participants implicitly critiqued the time and task model, which is broadly the system currently utilised. They discussed that there was not enough time for formal carers to provide high-quality care, which was frequently limited to basic physical care, did not allow for a meaningful relationship to develop and was not conducive to a condition such as dementia. At times, the consequence of this was that family members (who could be elderly or managing their own health problems) were tasked with either assisting the formal carer themselves or supporting the individual in ways they found challenging:

No not enough time. People with dementia are slow to understand and respond to questions and prompts to go to the toilet or to eat and drink. There is not enough time to properly assist often leaving me to do much of the work after they have left.

The night Carers were with us from 9.30 to 7.30 which was plenty of time. The morning Carers never came on time so I stopped them. The evening Carers rarely came at the time they were booked for, so I stopped them as well. They were meant to give me a break, but it was more stressful not knowing when they were coming so I just got on with things that needed doing by myself. Again, they were very task orientated.

More home care is needed as the disease progresses the family has no other option than to resort to a care home facility where if there was more home support the person with dementia could remain in their own home for longer and therefore freeing up valuable NHS services.

Similarly, some felt that systemic failings meant that there was not enough time for families to be included in important decisions, including assessments and care plans. This led to families feeling sidelined and, in some cases, meant that they were not only tasked with taking on an informal carer role but also as a result reported losing their personal relationship with their loved one. Some participants interpreted these failings as a dissonance between eligibility assessments, entitlement and availability (determined by the LA or hospital assessors) and the assessments of care needs as experienced by the informal carers:

Care arranged through social services is determined by cost. It’s not based on need or accurate assessment of need.

The most important factor is continuity of carers and close liaison with the rest of the family. I’ve approached the council re the frequent changes of carers but they say it’s because of budgetary restraints and shortage of home carers within the organisation.

I currently share the carer’s role but need also to maintain and run the family home. Being able to visit as 'The Daughter' just never happens.

Conversely, successful collaboration between formal and informal caregivers was perceived as a vital component of good care as this allowed for the needs of family units as a whole to be considered. Participants often referred to the importance of systems that allowed formal carers to spend time getting to know families to enable them to share their valuable insights into the service user:

These carers need to be given time to talk with both the person with dementia and their families. Home carers are coming into someone's home and being given responsibility for someone at the end of their life and they have the opportunity at that time to make it good or bad. Also, how they treat this person can affect their families forever bringing either joy or deep, deep sadness and guilt for letting strangers anywhere near their loved ones.

Good joint care planning. Needs assessment based on the family units’ needs not individual members separately. Small number of regular carers. Timeslots that are realistic and not rushed.

 4*. The importance of ongoing collaboration and care planning* was often reported as essential. Families and informal carers frequently stated a desire to collaborate with formal carers and assessors in the development and maintenance of care plans and the coordination of care, which they believed would be beneficial to the service user, the carer and the family unit. Care plans were often reported by participants as inadequately detailed, inflexible, uninformative, misused or not used at all. Again, this was linked to discontinuity of carers as well as problems with care coordination and systemic failings, which made it difficult for formal carers to collaborate to update the care plan consistently and accurately:

One of the senior managers at the agency came to do an assessment when they took on the contract. It was a pretty rushed affair and standard, as they tend to be; care plans don't get at the 'heart of the matter'. What the small but important care needs are that make all the difference.

Short frequent calls by many different carers no time to read care plan and get work done.

The care plan is fine as an outline for what needs doing, but on a daily basis needs to be adaptable.

For some, however, it was acknowledged that where care plans were used effectively and inclusively to collaborate with families/informal carers, this was beneficial for all in supporting the person with dementia:

Yes the carers that care for my mum are very good, very understanding. When the carers first came to help mum I was their first point of contact as I'm her main carer and you are given a diary and a knowing me paper to fill out. This helps the carers recognise mum and who she is.

Working collaboratively with me to look for solutions to manage Mum's incontinence.

## Discussion

Little research has explored formal home care for people with dementia, as experienced by their informal carers/families. However, the findings have highlighted the need for investment into meaningful personalisation, recognising the value of providing care and valuing formal carers, systemic failings of care coordination and provision and the importance of ongoing collaboration and care planning. These findings have echoed those of existing studies and have also provided further insight.

Research into the continuity of care for such individuals living at home is scant (Larsen et al., 2019), and so the present study has added to this literature and highlighted the importance of meaningful interaction between the person with dementia and their carer, which was highly valued by families. This was vital and should not solely focus on the functional aspects of care, allowing for communication and connection at a meaningful level. This is particularly crucial for those with dementia, where communication and language skills deteriorate; the condition is fluid and changeable and thus familiarity with carers is essential (e.g. Basting, 2009; Larsen et al., 2019; Osman et al., 2016). Findings also illuminated the difficulties formal carers faced in achieving such meaningful personalisation, where poor care coordination resulting in a discontinuity of carers for the individual leads to fleeting and disrupted relationships, meaning carers did not get to know their service users. Elsewhere, healthcare research has specifically highlighted the potential of the relational element of continuous care in maintaining trust and enhancing information flow (Haggerty et al., 2013; Larsen et al., 2019). Participants here largely reported a lack of continuity and experienced a lack of familiarity, generating further demand upon facilitating care for the individual, also reflecting previous research (Larsen et al., 2019).

Meaningful personalisation also pertains to cultural, emotional and social sensitivity (Milne & Smith, 2015). Participants here frequently cited this and highly valued this. This did not, however, only rely on the provision of adequate time to engage with the individual but also on efforts of the formal carer to provide this. Within dementia care, it is vital that the individual is seen as a person with feelings, emotions and a lifetime of personal experience. Food and music, for example can be essential for maintaining cultural identity for individuals with dementia (Anthony, 2015; Osman et al., 2016), which was reflected in the present study. Furthermore, experiences of dementia care can vary between different families, social groups and cultures and so efforts should be made to accommodate diversity in dementia homecare provision (Hamad et al., 2017).

Recognising the value of providing care and of valuing formal carers was essential in two ways. Firstly, carers who showed personal qualities and skills related to kindness, empathy, warmth and so on were as highly prized by informal carers and families as those with plentiful qualifications, as they were perceived as knowing the true familial value of providing care to their loved ones. This reiterates the importance of value-based care or care that is grounded around person centeredness (Davies et al., 2020; Veselinova, 2013). It demonstrates the importance of developing trust in formal carers to look after the person with dementia. The quality of care delivered not only determined the level of burden that was experienced by the informal carer/family (both emotionally in relation to guilt and physically in relation to assisting the formal carers) but also determined the individual’s day-to-day experiences and general well-being. Family burden is well documented (see Mosquera et al., 2016) and has been cited as increasing with a lack of continuity of care and as increasing the likelihood of a move to nursing home care (Bastawrous, 2013; Larsen et al., 2019). The present study reiterated this and further illuminates the meaning of burden to informal carers.

Secondly and linked to this, care work was perceived as undervalued and under-recognised broadly within society, resulting in systemic failings in relation to employment, training, contracts and wages, care coordination, rushed visits (based upon a time and task model of care) and poor care planning. The findings demonstrated that all of these factors were interlinked, as the time and task model of care in combination with the discontinuity of carers did not facilitate adequate time to learn about the person’s needs, collaborate with families and informal carers and to amend and update detailed care plans. The time and task model is a managerial framework adapted from the former National Health and Community Care Act (Department of Health, 1990), which aims to focus on the completion of care tasks within a designated time frame. In enacting this model, formal carers have frequently cited a lack of time to perform their care duties as well as write in care plans, choosing to ‘act rather than write’ (Larsen et al., 2019, p856). Informal carers have also previously reported a lack of adequate hours and flexibility in care delivery (McCabe et al., 2016). Moreover, commissioners of home care have themselves expressed the rushed nature of care visits due to budgetary constraints, the time and task model and the lack of status related to the care role, as devaluing the care role and as a barrier to recruitment and retention (Davies et al., 2020). Carers are also often coordinated via changeable rota systems and so continuity of care is not facilitated, which creates difficulties for carers in developing relationships with service users. Their precarious contracts also impact adversely upon continuity and the quality of care, which echoes international literature (Denton et al., 2015; Zeytinoglu et al., 2015). Additionally, participants alluded to a degree of distress experienced by formal carers as they were faced with a lack of resources and challenging situations which inhibited their ability to provide compassionate care. This resonates with growing literature regarding moral distress or injury in caregiving (Mänttäri-van der Kuip, 2016) and so should be investigated further. The restraints of the time and task model and inadequate budgets illuminate perceptions of carers as undervalued and underpaid and as reflecting a widely perceived crisis in the care workforce, also reflected in previous research (see Car et al., 2017; Cunningham et al., 2019). Participants’ perceptions of a lack of dementia-specific training were again linked to this undervaluing of and underinvestment into care work. Others have cited an inability of providers to ring-fence time for training and development of carers and a lack of care staff engagement with e-learning as problematic (Clarkson, et al., 2017; Cunningham et al., 2019). Moreover, Alzheimers UK (2016) have reported that 1 in 3 carers have no dementia training and families often feel staff have low learner confidence, inadequate education and limited qualifications (Cooper et al., 2017). Recognising the investment needed into the development of the care workforce, the restraints of the time and task model and acknowledging the value of care work more broadly may be central to the plight in developing the care workforce.

Furthermore, these systemic problems impacted negatively upon collaboration with informal caregivers and care planning generally within this study. Care planning was often inadequate or misused and collaboration was minimal, as plans often lacked detail and were not read properly. Conversely, where increased collaboration and consistent updating and reference to care planning were achieved, this was reported positively by participants. This tended to be within privately funded care and where this was not present was perceived as related to a dissonance between the LA and/or care provider assessment (those who assess need and develop the care plan initially) and the actual reported need as experienced by the service user or their informal carers. Those living with dementia and informal carers should be integrally included within care planning to promote optimally personalised formal care and to build trusting relationships (Karlsson et al., 2015). Participants in this study frequently reported the importance of collaborative care planning that reflected the individual’s personal preferences, needs and routines as well as the fluidity of their condition. This echoes previous research, reiterating that collaboration with close others is crucial within dementia care, where relationship-based care and a positive social environment have been specifically cited as beneficial (Molony et al., 2018). Ongoing relationships between formal and informal caregivers of people with dementia via oral collaboration and continuous care conducted in this manner have also been noted as improving care. Such collaborations simultaneously limit bureaucracy, minimise the sole reliance upon written care planning documentation and facilitate a more person-centred approach as relationships are built and developed and a richer understanding of the person is acquired (Larsen et al., 2019). The findings here demonstrate that systemic constraints did not allow for consistency in utilising written care records or for ongoing relationships and oral collaboration.

### Implications for policy

1. The UK Government’s Dementia 2020 Challenge Review (2019) has highlighted that the objectives of achieving meaningful care for those living with dementia are some way from being met and this study has echoed this.
2. Standards of care greatly vary across England and Wales and access to consistent, high-quality formal dementia care is still experienced by families and informal carers as inadequate.
3. Valuing providing care and valuing carers may be crucial to reducing this burden and improving the well-being of the person with dementia, which may be pivotal in reducing the likelihood of a move to a nursing home for the individual.
4. This study highlights the benefits of ongoing informal carer inclusion in personalised care planning and care coordination for those living with dementia. However, current definitions and practices related to home care predominantly focus upon supporting function and are time- and task-oriented and do not facilitate optimal care provision that supports the informal carers or overall health and well-being of the person with dementia. The appropriateness of the continued use of the time and task managerial model of care, particularly for people with dementia should be urgently reviewed.
5. Barriers already identified include a transient workforce and budgetary demands. The present study echoes this and points to potential solutions that lie within a system that allows for close, ongoing, adaptable collaboration and care planning between formal and informal carers.
6. Acknowledgement in policy of the highly valued, highly skilled and intricate nature of dementia care is necessary to ensure that dementia care and care work more broadly is recognised for the immense societal contribution it provides.

### Implications for education/training

1. Investment into the education and training of carers, particularly for those who provide specialised care to people with dementia is urgently required.

### Implications for practice

1. Continuity of care and familiarity of formal carers is key to understanding the complexities of living with dementia and to developing meaningful relationships, both with the individuals and with their informal carers and/or families.
2. Equally, cultural, social, emotional sensitivity and maintaining personhood should be at the heart of high-quality dementia home care. A lack of such relationships and care is inadequate for people with dementia and may generate further burden upon informal carers and subsequently generate further demand on facilitating care.

### Strengths and limitations of the study

The study’s main strengths are that it was co-designed by informal carers and asked for detailed, anonymous, data on people’s views of home care for dementia, which is a neglected and under-researched service in the United Kingdom. It was limited in that it was only a point in time survey. The provision of home care is fast changing, and government reforms are looking at home care and the rest of social care now as an element of reform. It was not possible to collect very specific information as the survey was intended to be completely anonymous and not to collect any personal data.

## Conclusions

When considering the original research question of ‘what is appropriate home care for people with dementia and how does it fit with the preferences of informal carers?’ it is highly personalised and meaningful care, accessible within a system which facilitates time and consideration to be taken for ongoing collaboration between formal and informal carers. The preferences of informal carers are largely for care to be, continuous with a formal carer, carefully deliberated, personalised and fluid in adapting to the changing needs of the person with dementia and the individuals who support them. This will require combined efforts from policymakers and care providers to recognise the value and intricate nature of caregiving by investing in workforces and acknowledging the potential and value of both formal and informal caregivers in dementia home care. This can only be achieved by working with all stakeholders to tangibly implement as standard within care, the importance of establishing and maintaining meaningful relationships for those living with dementia and their informal carers.
